# Changes of cell growth and magnetosome biomineralization in *Magnetospirillum magneticum* AMB-1 after ultraviolet-B irradiation

**DOI:** 10.3389/fmicb.2013.00397

**Published:** 2013-12-19

**Authors:** Yinzhao Wang, Wei Lin, Jinhua Li, Yongxin Pan

**Affiliations:** ^1^Biogeomagnetism Group, Paleomagnetism and Geochronology Laboratory, Key Laboratory of the Earth's Deep Interior, Institute of Geology and Geophysics, Chinese Academy of SciencesBeijing, China; ^2^France-China Bio-Mineralization and Nano-Structures Laboratory, Chinese Academy of SciencesBeijing, China

**Keywords:** magnetotactic bacteria, ultraviolet-B radiation, cell growth, microbial biomineralization, rock magnetic measurement

## Abstract

Effects of ultraviolet radiation on microorganisms are of great interest in field of microbiology and planetary sciences. In the present study, we used *Magnetospirillum magneticum* AMB-1 as a model organism to examine the influence of ultraviolet-B (UV-B) radiation on cell growth and magnetite biomineralization of magnetotactic bacteria (MTB). Live AMB-1 cells were exposed to UV-B radiation for 60, 300 and 900 s, which correspond to radiation doses of 120 J/m^2^, 600 J/m^2^, and 1800 J/m^2^, respectively. After irradiation, the amounts of cyclobutane pyrimidine dimers (CPD) and reactive oxygen species (ROS) of the cells were increased, and cell growth was stunted up to ~170 h, depending on the UV-B radiation doses. The UV-B irradiated cells also produced on average more magnetite crystals with larger grain sizes and longer chains, which results in changes of their magnetic properties.

## Introduction

Solar ultraviolet radiation (wavelengths 10-400 nm) fluctuation is proposed as one of the most important selective pressures on the evolution of life on Earth (Cockell, 1998; Garcia-Pichel, 1998; Häder et al., 2007; Sinha and Häder, 2008; Weigand and Sundin, 2012). Although the stratospheric ozone protects organisms on Earth from UV radiation exposure (Solomon, 1999), the ozone layer can be stripped away by solar wind when the strength of the Earth's magnetic field is low. For instance, during geomagnetic excursion or polarity reversal, the field is thought to 20% of its typical strength (Reid and Isaksen, 1976; McKenzie et al., 2003; Glassmeier and Vogt, 2010; Stadelmann et al., 2010; Valet and Valladas, 2010). Consequently, the depletion of the ozone layer will lead to increased penetration of harmful radiation to the Earth's surface, which may affect aquatic ecosystems (Hargreaves, 2003; Wulff et al., 2008). UV-C radiation (wavelengths 10–280 nm) can normally be absorbed by the ozone layer, but UV-B (wavelengths 280–320 nm) and UV-A radiation (wavelengths 320–400 nm) can reach the biosphere and influence living organisms (Zepp et al., 1998; Zenoff et al., 2006; Konhauser et al., 2007; Santos et al., 2013). In addition to enhancing production of intracellular reactive oxygen species (ROS) (similar to the UV-A radiation), UV-B radiation could directly damage DNA in organisms and thus should be more detrimental to organism health (Qiu et al., 2005b; Hernandez et al., 2006; Santos et al., 2013).

Microorganisms are the earliest known species to have appeared on Earth. Various microorganisms could form minerals and thus can leave fossil records that have been used to trace early life on Earth and other planets such as Mars (Li et al., 2013a). However, how UV radiation influences the microbial biomineralization and the formation of fossil biominerals or mineral-encrusted cells (i.e., microfossils) is still poorly understood (Cockell, 1998; Garcia-Pichel, 1998; Häder et al., 2007; Singh et al., 2010). Recent investigations on aquatic bacterial isolates have revealed that UV-B radiation could lead to DNA lesion and cellular oxidative stress (Agogué et al., 2005; Santos et al., 2011, 2012, 2013; Matallana-Surget et al., 2012; Singh et al., 2012). On the other hand, some microorganisms have evolved strategies to mitigate such irradiation damage (Sinha and Häder, 2008; Schmidt et al., 2009; Weigand and Sundin, 2009; Singh et al., 2010; Weigand et al., 2011). For example, *in vitro* biomineralization of iron-silica biominerals of filamentous cyanobacterium *Calothrix* help the bacteria efficiently coping with UV irradiation. Compared with non-mineralized bacteria, the mineralized bacteria represent remarkable resistance to UV irradiation as revealed by the rates of photosynthesis, chlorophyll-a content, and phycocyanin autofluorescence (Phoenix et al., 2001). Cyanobacterium *Lyngbya majuscula* was found to produce chemical compounds that were able to absorb UV radiation and synthesize an incrassate cell sheath under enhanced UV-B irradiation (Mandal et al., 2011).

Magnetotactic bacteria (MTB) are able to synthesis single domain (SD) magnetic particles within intracellular membrane organelles called magnetosome (Bazylinski and Frankel, 2004; Faivre and Schüler, 2008). The magnetosome, usually arranged in chain(s), facilitate MTB to navigate along the Earth's magnetic field and efficiently find the optimal living conditions in aquatic and sedimentary environments, a process known as magnetotaxis (Bazylinski and Frankel, 2004; Pan et al., 2009). After MTB die and are buried, the magnetosome crystals can be preserved as fossil biominerals (magnetofossils), which are potential geological records for paleoenvironmental and paleomagnetic information, as well as biosignatures for searching early life on Earth and extraterrestrial life (Petersen et al., 1986; Chang and Kirschvink, 1989; McKay et al., 1996; Yamazaki and Kawahata, 1998; Arató et al., 2005; Pan et al., 2005; Kopp and Kirschvink, 2008; Paterson et al., 2013). Previous studies have demonstrated that environmental factors, such as salinity, temperature, the strength of geomagnetic field, oxygen and iron resource, can influence the overall diversity of MTB communities in nature and the compositional, physical, and magnetic features of magnetosomes (e.g., Lins et al., 2007; Faivre et al., 2008; Lin et al., 2011, 2013a,b; Li and Pan, 2012; Wang et al., 2013). However, to our knowledge, the influence of UV radiation on MTB has not been studied yet, although it is of particular interest for better understanding the evolution of magnetotaxis in early history of Earth. During a geomagnetic excursion or polarity reversal, where the dipolar geomagnetic field was weak and the UV radiation was strong, how MTB respond to the environmental changes also remains unknown. In the present study, we have investigated the effects of UV-B radiation on cell growth and magnetite biomineralization of *Magnetospirillum magneticum* AMB-1. Possible relationships between magnetosome formation and UV-B irradiation are discussed.

## Materials and methods

### Preparation of cell and growth conditions

A facultative anaerobic MTB strain *Magnetospirillum magneticum* AMB-1 (ATCC700264) was used in this study. In all experiments, cells were cultivated in the magnetic spirillum growth medium (MSGM) (Blakemore et al., 1979). This medium (1 L) contains 5 ml of Wolfe's mineral solution, 10 ml of Wolfe's vitamin solution, 0.68 g of potassium phosphate, 0.12 g of sodium nitrate, 0.05 g of sodium acetate, 0.035 g of ascorbic acid, 0.37 g of tartaric acid, 0.37 g of succinic acid, and 50 μ M of ferric quinate. In order to obtain a uniform genotype strain, originally activated AMB-1 cells were plated onto MSGM agar plate for 7 days, single clone was picked up and enlarged cultivated for the following studies. Non-magnetic AMB-1 cells were obtained by growing cells under 220 rpm rotation at 26°C with free air exchange. The non-magnetic cells were then inoculated equally to twelve UV-B penetrable quartz bottles. Each bottle contained 300 mL of MSGM culture medium and was incubated under the same cultivation condition. When the cell density reached ~1.0 × 10^7^ cells/ml at early exponent phase, the bottle was used for the irradiation experiment.

### Source of UV-B radiation and irradiation experiment

**Figure 1:**
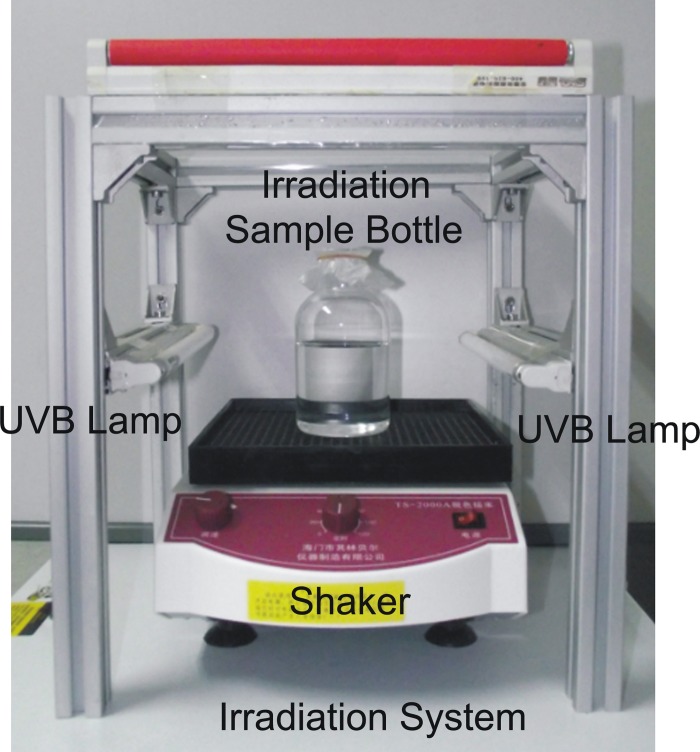
**Double-sided UV-B irradiation device.** The high purity quartz irradiation bottle is designed with flat bottle well sitting on a shaker to provide a uniform irradiation environment.

The irradiation experiment system is shown in Figure 1. UV-B radiation was obtained from USHIO ultraviolet lamps (USHIO America, USA). The lamps were coated with special blended phosphor to emit radiation peaking at 306 nm. The radiation intensity of light source was measured by a double-channel UV-B radiometer with a detective range between 275 and 330 nm (Beijing Normal University, China). The double-sided UV-B irradiation system with an averaged UV-B radiation intensity of 2.0 W/m^2^ was used to carry out the experiment. The irradiation bottles containing AMB-1 inoculations were exposed to UV-B radiation for 60, 300, and 900 s, which were equivalent to radiation doses of 120, 600, and 1800 J/m^2^, respectively. To ensure even exposure the bottles were rotated at 50 rpm during irradiation. Non-irradiated cell samples (bottles covered with aluminum foils) were taken as experimental control. All twelve irradiation bottles with AMB-1 cells were divided into the four different experiment groups with tripled repeats of each UV-B radiation condition.

After the irradiation, 10 mL of cells were immediately taken from the bottle for cyclobutane pyrimidine dimers (CPD) and ROS analyses. The rest of the culture was centrifuged at 4°C 6000 rpm for 10 min. Then the cells of each sample were washed by 0.9% NaCl (wt/vol) and inoculated into 500-mL fresh MSGM medium, respectively under anaerobic condition in the dark. The cell growth of each group was examined through the optical density at 600 nm (OD_600_). As the sample reached stationary phase for 40 h, the cells were harvested for cell morphology, magnetosome crystal, and magnetic analyses.

### Determination of relative CPD production

The relative CPD production was used to assess the DNA damage in the UV-B irradiated cells. The DNA was extracted using TIANamp Bacteria DNA Kit (TIANGEN, China). The concentrations of DNA were determined by the absorbance at 260 nm and calculated by Lambert-beer law. In order to generate a uniform comparison, each DNA sample was diluted to 1 μ g/mL. For enzyme-linked immunosorbent assay, first, 0.04% protamine sulfate was coated to a polyvinylchloride flat-bottomed microtitre plate for 2 h at 37°C. Fifty microliters of the denatured DNA were put into the plate wells and incubated for 1 h at 90°C. The plate was washed 5 times by phosphate-buffered saline with twain (PBS-T) after drying. Then 200 μ L of 3% BSA were add to each well and incubated at 37°C for 30 min and washed as described above. After blocking, 100 μ L anti-thymine dimer antibody [H3] (abcam, USA) were added and incubated at 37°C for 1 h, then washed again. The plate with 100 μ L/well HRP affinipure goat anti-mouse IgG (EarthOx, USA) was incubated at 37°C for 30 min. Then, the plate was washed 3 times with PBS-T and 2 times with citrate-phosphate buffer of pH 5.0. Finally, 100 μ L of o-phenylene diamine and 0.007% hydrogen peroxide in citrate-phosphate buffer were then added to each well and incubated for 30 min at 37°C. Fifty microliters of 2 mol/L H_2_SO_4_ were added to stop the reactions. The absorbance was then read at 490 nm using a microplate reader (SynergyTMH4, BioTek, USA) to determine the CPD photoproduct. The relative generations of CPD were normalized by the absorbance of the blank control.

### Detection of ROS generation

The generation of ROS was analyzed by using DCFH-DA method based on previous report (He and Häder, 2002). DCFH-DA can be taken by cell and hydrolyzed to DCFH, which can not pass through cell membranes and is trapped within cell. Intracellular ROS can oxidize nonfluorescent DCFH into DCF, a highly fluorescent compound. Here, after irradiation, 2 mM 5 μ L DCFH-DA were immediately added to 200 μ L of the irradiated samples and incubated at 37°C for 30 min in dark. The fluorescence of the samples was measured using a spectrofluorometer (SynergyTMH4, BioTek, USA) with the excitation wavelength of 488 nm and emission wave lengths between 500 and 600 nm. The fluorescence intensity at 525 nm, after subtraction of the fluorescence of blank control then normalizing to the protein content, was used to determine the relative ROS contents. Protein content was obtained by BCA protein assay kit (Thermo Scientific, USA).

### Cells and magnetosomes analyses

Morphologies of cells and magnetosome crystals of irradiated AMB-1 were analyzed using a JEOL JEM-1400 transmission electron microscope (TEM) with an accelerating voltage of 80 kV. About 20 μL of cells were dropped onto a copper TEM grid covered with carbon-coated formvar film for 2 h, then washed twice with sterilized distilled water and dried in air. Magnetosome sizes were defined as (length + width)/2, and the shape factors as width/length by measuring TEM micrographs.

For rock magnetic measurements, the cells were harvested by centrifugation at 4°C. To prevent possible oxidation, the cell pellets were immediately transferred into a COY anaerobic chamber [(O_2_) < 300 ppm, COY-7000220A, USA] and loaded into non-magnetic gelatin capsules and dried for overnight. The samples were stored in −20°C with N_2_ protection prior to measurements (Li and Pan, 2012). Room temperature magnetic measurements were performed on a Model 3900 vibrating sample magnetometer (Princeton Measurements Corporation, USA, sensitivity 5.0 × 10^−10^ Am^2^). Hysteresis loops were measured between ± 300 mT in 3 mT increments with 500 ms averaging time. The hysteresis parameters saturation magnetization (*M*_*s*_), saturation remanence (*M*_*rs*_), and coercivity (*B*_*c*_) were determined after paramagnetic correction. Remanence coercivity (*B*_*cr*_) was determined from a back-field demagnetization of the saturation isothermal remanent magnetization (SIRM) acquiring at 1.5 T field. First-order reversal curves (FORCs) were measured and calculated using the FORCinel version 1.22 software (Roberts et al., 2000; Harrison and Feinberg, 2008; Egli et al., 2010). The median coercivity (*B*_*c*, FORC_) and half-width interaction field (*B*_*b*, 1/2_) were calculated (Muxworthy and Dunlop, 2002; Winklhofer and Zimanyi, 2006; Li et al., 2013b).

Low-temperature magnetic measurements were performed on a Quantum Design Magnetic Property Measurement System (MPMS XP-5). Zero-field cooling (ZFC) and field cooling (FC) curves were acquired by first cooling the samples from 300 to 10 K in a zero field and in a 2.5 T field, respectively, and then measuring the demagnetization of remanence in zero field during warming from 10 to 300 K. The Verwey transition temperature (*T*_*v*_) were determined as the temperature for the maximum value of the first derivative of the FC curves. The δ-ratio (δ_FC_/δ_ZFC_), where δ = (*M*_80*K*_ − *M*_150*K*_)/*M*_80*K*_, were calculated according to Moskowitz et al. (1993).

## Results

### The cell growth after UV-B irradiation

**Figure 2:**
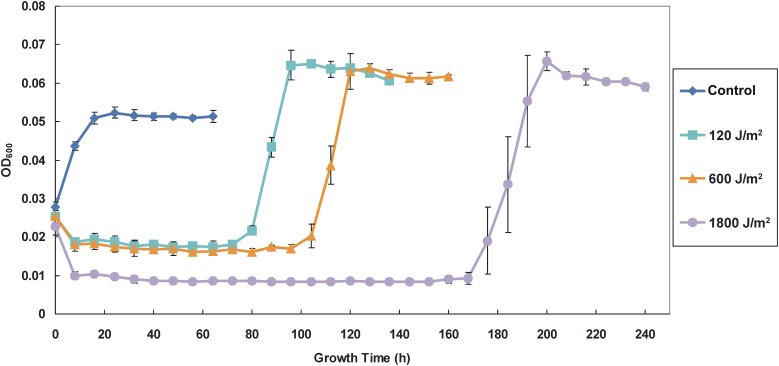
**Growth curves of the control (non-irradiated) and the irradiated AMB-1 cells.** The diamond, square, triangle and circle shaped lines indicate the growth density (determine as OD_600_) of the control and irradiated cells after 120, 600, and 1800 J/m^2^ irradiation.

The effect of UV-B radiation on cell growth indicates a clear dose dependence as revealed by OD_600_ (Figure 2). The initial OD_600_ values of all samples were about 0.025 after re-suspending the irradiated cells in fresh medium. After the first 8 h following re-suspension, the cell concentrations of the 120, 600, and 1800 J/m^2^ irradiated samples slightly decreased to 0.019, 0.018, and 0.01, respectively, which indicates differing degrees of cell density loss. Subsequently, before they reached exponent growth phases and stationary phases, the irradiated samples remained relatively stable at low levels cell density (static phase or recovery time) for 72, 96, and 168 h for the groups received 120, 600, and 1800 J/m^2^ irradiation, respectively. The non-irradiated control group exhibited rapid growth after re-suspension and reached constant at stationary phase within 24 h. We also note that the 1800 J/m^2^ irradiation group had the longest recovery period and the largest standard deviation during growing at exponent phase, which suggests that the higher dose of irradiation might have caused more damage to cells in this group. Compared with the control group, all irradiated groups had a relatively higher OD_600_ in the stationary phase after recovery.

### Variations of the CPD formation and the ROS accumulation

**Figure 3:**
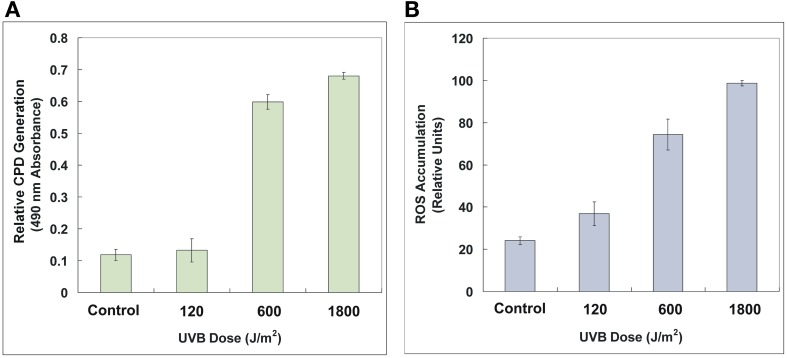
**Relative CPD generation (A) and ROS accumulation (B) of the control and the irradiated AMB-1 cells.** The CPD formation was determined by 490 nm absorbance. The ROS content was characterized by 525 nm fluorescent intensity normalized protein content. See text for details.

As expected, UV-B radiation caused significant deleterious effects on DNA and cellular levels that correspond to the generation of CPD and the accumulation of ROS, respectively, which also exhibited dose-dependent patterns (Figure 3). CPD, induced by UV-B photons, is a photoproduct known as DNA lesions, which can blocks DNA replication and transcription. ROS is a collection of superoxide radical (O^·−^_2_), hydroxylradial (OH^·^), hydrogen peroxide (H_2_O_2_) and singlet oxygen (^1^O_2_) (He and Häder, 2002). These free radicals can lead to damages of lipids, proteins, and DNA. In our experiments, the strain AMB-1 exhibited distinct detrimental effects under different levels of UV-B irradiation. Specifically, for the control, 120, 600, and 1800 J/m^2^ groups, the amount of relative CPD generation were 0.118 ± 0.018, 0.133 ± 0.037, 0.599 ± 0.023, and 0.680 ± 0.011, while the ROS value reached 24.07 ± 1.79, 36.81 ± 5.58, 74.33 ± 7.37, and 98.66 ± 1.22 (determined by normalized relative units), respectively.

### Changes of the cell size, magnetosomes and chain structures

**Figure 4:**
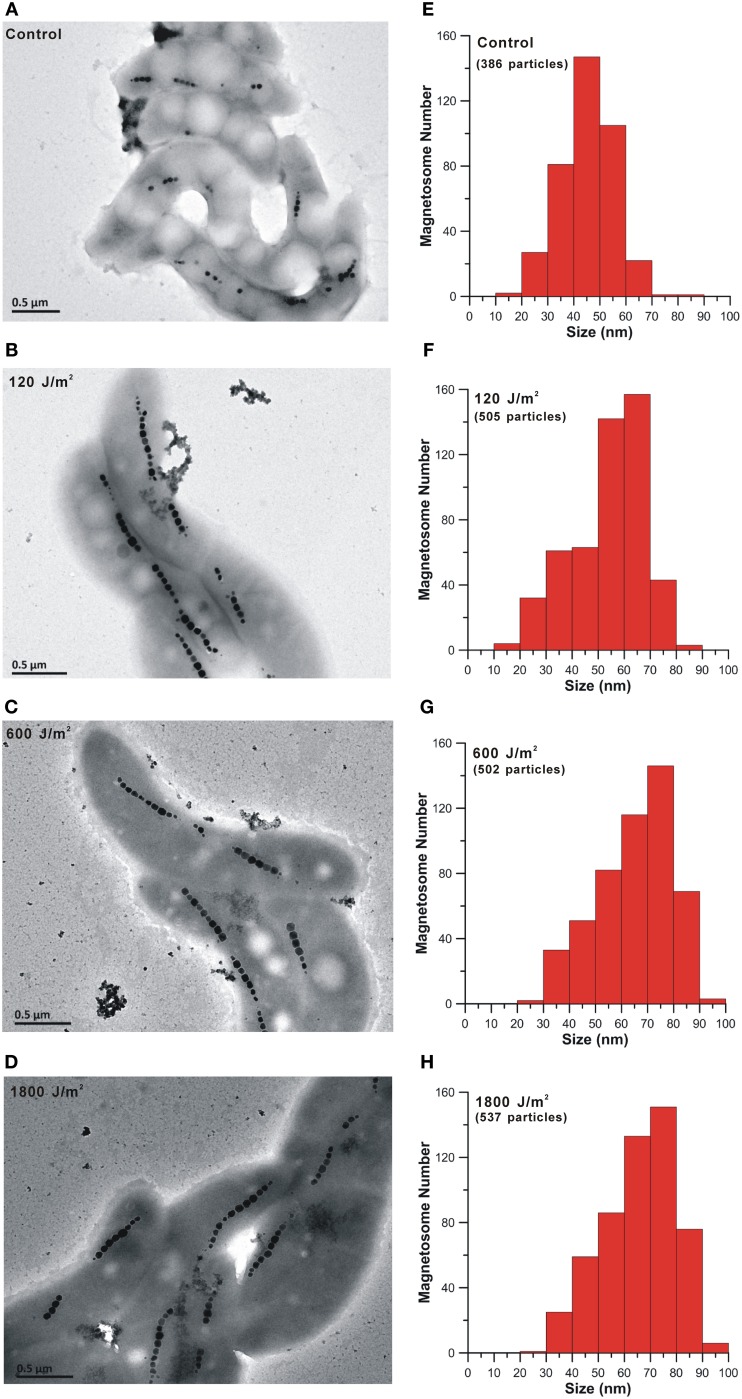
**TEM micrographs of cell morphology and magnetosome chains and histograms of magnetosome grain sizes of the control (A and E) and the irradiated AMB-1 cells (B–D and F–H)**.

The detailed information of the AMB-1 cells and magnetosomes after the bacteria recovered from UV-B irradiation are summarized in Table 1. Figures 4A–D are representative TEM micrographs of the AMB-1 cells of the four experiment groups. Interestingly, all UV-B irradiated cells produced larger grain sizes and longer magnetosome chains compared with the non-irradiated cells. Statistic analyses of each group indicate that the magnetosome grain size of the irradiated cells was nearly 10 nm larger on average than the non-irradiated cells. For the irradiated groups, we noticed decreased number of subchains per cell (*N*_*s*_), increased number of magnetosomes per cell (*N*_*m*_) and per subchain(*N*_*sm*_) (Table 2). However, the average cell lengths of the irradiated cells were about 0.4 μm shorter than the control group. Although all irradiated samples had differences in cell size and magnetosome parameters, no clear dose-related change trend was observed.

**Table 1:** **Cell length, magnetosome, and subchain information of the AMB-1 samples at different irradiation levels**.

**UV-B dose (J/m^2^)**	**Cell length (μ m)**	**Magnetosome**			**Magnetosome chain**	
		**Size (nm)**	**Shape factor**	***N*_*m*_ (per cell)**	***N*_*s*_ (per cell)**	***N*_*sm*_ (per subchain)**
Control	3.01 ± 0.41	45.44 ± 10.31	0.913 ± 0.077	11 ± 3	3 ± 1	3 ± 2
120	2.64 ± 0.65	54.24 ± 13.84	0.860 ± 0.088	20 ± 6	2 ± 1	10 ± 4
600	2.56 ± 0.38	55.43 ± 14.43	0.857 ± 0.089	19 ± 5	2 ± 1	9 ± 3
1800	2.61 ± 0.43	55.79 ± 13.78	0.859 ± 0.085	21 ± 5	2 ± 1	10 ± 4

**Table 2:** **Magnetic parameters of the bulk AMB-1 samples at different irradiation levels**.

**UV-B dose (J/m^2^)**	***B*_*c*_ (mT)**	***B*_*cr*_ (mT)**	***B*_*cr*_/*B*_*c*_**	***M*_*rs*_/*M*_*s*_**	***B*_*c*, FORC_ (mT)**	***B*_*b* 1/2_ (mT)**	***T*_*v*_ (K)**	**δ _*FC*_**	**δ _ZFC_**	**δ _*FC*_/δ _ZFC_**
Control	22.54	34.08	1.51	0.46	34.9	1.31	102	0.216	0.098	2.19
120	35.98	44.42	1.23	0.52	43.6	1.41	102	0.222	0.076	2.93
600	36.27	47.28	1.30	0.54	46.0	1.51	102	0.197	0.072	2.73
1800	34.41	43.25	1.26	0.52	43.4	1.41	102	0.159	0.044	3.61

### The coercivity, FORC diagrams and δ-ratios

**Figure 5:**
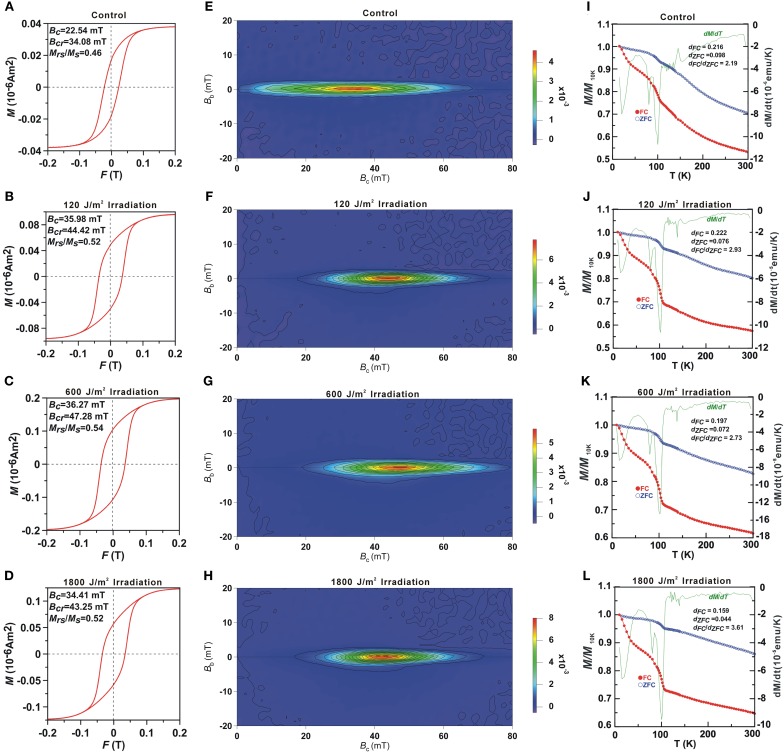
**Room temperature hysteresis loops (A–D), FORC diagrams (E–H) and low temperature magnetization curves (I–L) of the control and the irradiated cell bulk samples.** In **(I–L)** the red, blue and green lines represent the FC, ZFC curves and first derivative of FC curves, respectively.

Magnetic properties of the whole cell samples were displayed in Table 2 and Figure 5. Consistent with previous studies on whole-cell MTB samples, all samples were characterized by Stoner-Wohlfath-type hysteresis loops with *M*_*rs*_/*M*_*s*_ ratio close to 0.5 (Stoner and Wohlfarth, 1948), and central-ridged FORC diagrams characterized with a narrow distributed along *B*_*c*_ axis (Figures 5E–H), indicating non-interacting uniaxial SD assemblages (Egli et al., 2010). In comparison, the room temperature magnetic parameters *B*_*c*_, *B*_*cr*_ and, *B*_*c*, FORC_ of the UV-B radiation-treated samples were nearly identical, but about 10 mT higher than the control group. The decreased *B*_*cr*_/*B*_*c*_ values, increased *M*_*rs*_/*M*_*s*_ values and right-shifted FORCs suggest that the radiation treated samples possess magnetite crystals with larger grain sizes and/or longer chains, which have also been confirmed by the TEM observations (Figure 4).

Low-temperature measurements (Figures 5I–L) showed that the magnetic remanence of the UV-B radiation treated cells decreased rapidly at both ~15–30 and ~102 K in FC curves. The *T*_*v*_ of 102 K was similar to previous reports of magnetosome produced by strain AMB-1 (e.g., Li et al., 2009), which indicates no distinct change in the magnetosome composition by irradiation treatment. The δ -ratio of the control, 120, 600, and 1800 J/m^2^ irradiated samples were 2.10, 2.93, 2.73, and 3.61, respectively, which further demonstrates better magnetosome chain structures in the radiation treated groups (Moskowitz et al., 1993).

## Discussion

### The biological deleterious effects of UV-B irradiation on AMB-1 cells

In this study, *M. magneticum* AMB-1 was used as a model organism to probe the UV-B biological effects on the cell growth and biomineralization. We found an initial decrease of cell density (OD_600_) and a significant inhibition of growth of the AMB-1 after the bacteria were irradiated under doses of 120, 600, and 1800 J/m^2^ (Figure 2). The increased CPD formation and the ROS accumulation (Figure 3) indicated clearly UV-B dose-dependent deleterious effects on both DNA and cellular levels after UV-B irradiation. The self-repair after UV-B radiation might cause the observed prolonged static period of irradiated AMB-1 cells (Agogué et al., 2005; Goosen and Moolenaar, 2008). However, after the cells fully recovered (40 h after reaching stationary growth phases), the accumulations of CPD were 0.075 ± 0.007, 0.086 ± 0.027, 0.092 ± 0.036 and 0.088 ± 0.033, and the ROS values were 31.45 ± 5.48, 25.76 ± 3.56, 30.04 ± 8.31, and 30.50 ± 3.64 for the control, 120, 600, and 1800 J/m^2^ irradiated groups, respectively. Those values of the irradiated groups were comparable to the non-irradiated control group, which suggests that AMB-1 cells are able to repair the UV-B radiation damage.

Previous studies on repair strategies of aquatic organisms have demonstrated that after UV-B irradiation bacteria displayed a high efficient DNA light and dark repair to eliminate CPD that would block the replication and transcriptional process and lethiferous (Goosen and Moolenaar, 2008). For ROS that leads to cell membrane damage and cellular injuries, a series of antioxidant enzyme (e.g., superoxide dismutase, catalase, and peroxidase) can be induced to mitigate the negative effects caused by ROS (He and Häder, 2002; Xie et al., 2009; Santos et al., 2012). Transcriptome analysis of UV irradiated bacteria such as *Deinococcus* sp. and cyanobacterium *Synechocystis* also exhibited the up regulation of DNA repair and stress response-related genes. Both base/nucleotide excision repair and recombinational repair pathway participated in UV induced damage repair. Genes that were involved in scavenging oxygen radicals were also noticed up regulated after UV irradiation (Reid and Isaksen, 1976; Huang et al., 2002; Qiu et al., 2005a,b; Yuan et al., 2012).

Except for high efficiency removal of toxic oxygen species and specialized light and dark repair systems, other abilities (e.g., positive migration and production of UV screening pigments and compounds) can also help bacteria in avoiding harmful UV radiation (Ehling-Schulz and Scherer, 1999; Goosen and Moolenaar, 2008; Singh et al., 2010). Compared with non-magnetotactic bacteria in nature, MTB may migrate efficiently to deeper water layer or into sediments to avoid harmful UV radiation owing to their unique magnetotaxis. In addition, magnetite/greigite crystals may protect MTB from UV radiation.

### Possible effects on magnetosome biomineralization

TEM observations revealed that the irradiated cells, with a prolonged static phase, synthesized more and larger magnetosomes, as well as longer chains, which were further confirmed by various rock magnetic measurements. All irradiated samples had higher *B*_*c*_, *B*_*cr*_, *M*_*s*_, *M*_*rs*_ and δ-ratio, and the FORC diagrams exhibited right shifts. Increased *B*_*c*_, *B*_*cr*_ and *B*_*c*, FORC_ were largely due to the increased magnetosome chain lengths and grain sizes, while higher *M*_*s*_ and *M*_*rs*_ were probably caused by increased magnetosome production, consistent with previous studies on magnetite particles within the SD range (Kobayashi et al., 2006; Kopp and Kirschvink, 2008; Li et al., 2009). When we reinoculated these irradiated but fully recovered cells into the same fresh medium once more, however, no enhancement of magnetosome biomineralization was detected in the stationary phases compared with the non-irradiated group. This suggests that the observed enhanced magnetite biomineralization maybe the regulation induced by irradiation rather than a genetic imprint or radiation selection under the used experimental condition for AMB-1. Some self-repair processes and related metabolism changes may influence the crystallization of magnetosomes as well. At this stage, we cannot exclude that the prolonged recovery time of irradiated cells might affect the magnetite magnetosome formation.

Magnetite magnetosome biomineralization of MTB have been well documented by the molecular biologists and mineralogists (Bazylinski et al., 2007; Faivre and Schüler, 2008; Pósfai and Dunin-Borkowski, 2009). Previous studies have shown that biomineralization of chain arranged magnetite magnetosomes undergo several steps: formation of membrane vesicles on filamentary skeleton, iron uptake and magnetite crystallization within magnetosome membrane (Komeili, 2012). It has been found that the synthesis of magnetite magnetosome was closely related to nitrate reduction pathway (Bazylinski and Blakemore, 1983; Ge et al., 2011; Wang et al., 2011; Li et al., 2012). Iron and oxygen metabolism regulation also had significant influences on biomineralization of the cultured MSR-1 strain (Qi et al., 2012; Rong et al., 2012). Based on TEM micrographs, in addition to the magnetosome changes, the irradiated groups possess less round granules (possibly PHA which described by Keim et al., 2005; Silva et al., 2008) than the non-irradiated control group (data not shown). This is likely caused by the prolonged cell growth or some potential modification in the metabolism pathways within the irradiated cells. It also implies that the UV-B radiation may induce metabolism changes in AMB-1, which in turn affects magnetosome biomineralization. This suggestion is also supported by previous reports that UV radiation can strongly affect the metabolism of other microorganisms. For example, up regulated nitrate reduction genes such as *nap* and *nos* genes and genes related to iron-sequestering (*bpc* and *hem*H) and peroxide metabolism (e.g., *oxy*R, *kat*G, and *sod*B) were verified through transcriptome analyses of both radio-resistant bacterium *Deinococcus* species and a facultative anaerobic *Gammaproteobacteria Shewanella oneidensis* MR-1 after UV irradiation (Qiu et al., 2005a,b). Nevertheless, further investigations on time-series culture medium composition analyses and transcriptome of the irradiated AMB-1 cells are needed to reveal the mechanisms of these phenomena.

It is reasonable to assume that the enhanced biomineralization of magnetosomes in MTB may have some benefits for resistance of irradiation. The large size, number, and improved chain structures of magnetosomes in the MTB cell may greatly help bacteria to mitigate damage caused by UV-B. For example, the magnetosomes themselves are able to prevent the radiation and eliminate intracellular ROS (Guo et al., 2012). Moreover, the better magnetite magnetosome formation in cells may enhance the sensibility of magnetotaxis navigation to escape from UV exposure when magnetic field is low. A deep-branching group MTB, affiliating within the *Nitrospira* phylum, which contains a few hundred to a thousand bullet-shaped magnetite magnetosomes, are widely found in aquatic environments (Spring et al., 1993; Pan et al., 2005; Li et al., 2010; Jogler et al., 2011; Lin et al., 2012). Our new data support that the synthesis of large amount of magnetosomes within one cell might be significant evolutionary benefit for such MTB that lived on early Earth as the geomagnetic field was probably weak and the UV radiation was strong.

### Conflict of interest statement

The authors declare that the research was conducted in the absence of any commercial or financial relationships that could be construed as a potential conflict of interest.
