# Supplementary figures and images for: Changes of cell growth and magnetosome biomineralization in Magnetospirillum magneticum AMB-1 after ultraviolet-B irradiation
Source: Front Microbiol. 2013 Dec 19;4:397. doi: 10.3389/fmicb.2013.00397 (PMC3867805; doi:10.3389/fmicb.2013.00397)

**0 J/m<sup>2</sup>**

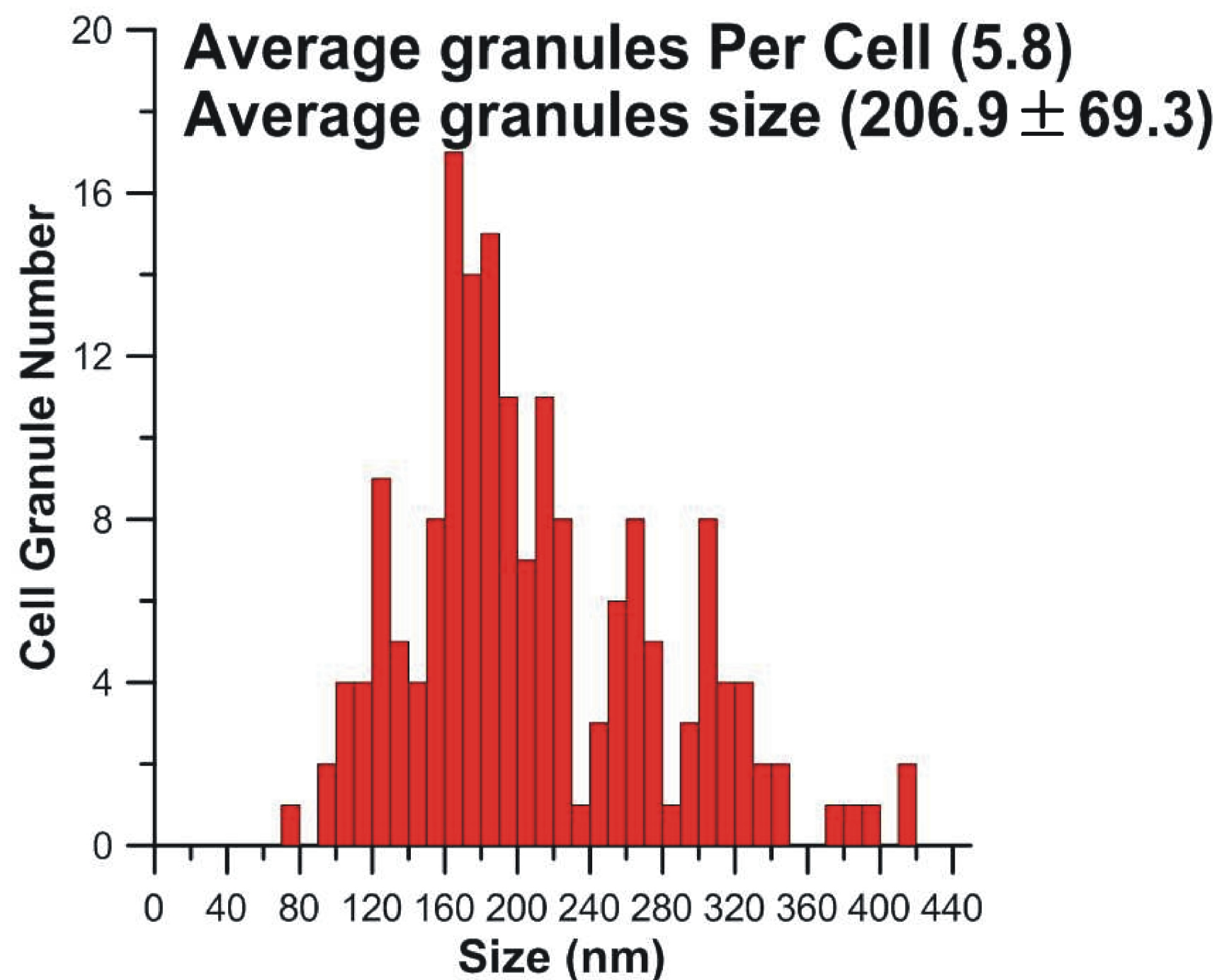

**120 J/m<sup>2</sup>**

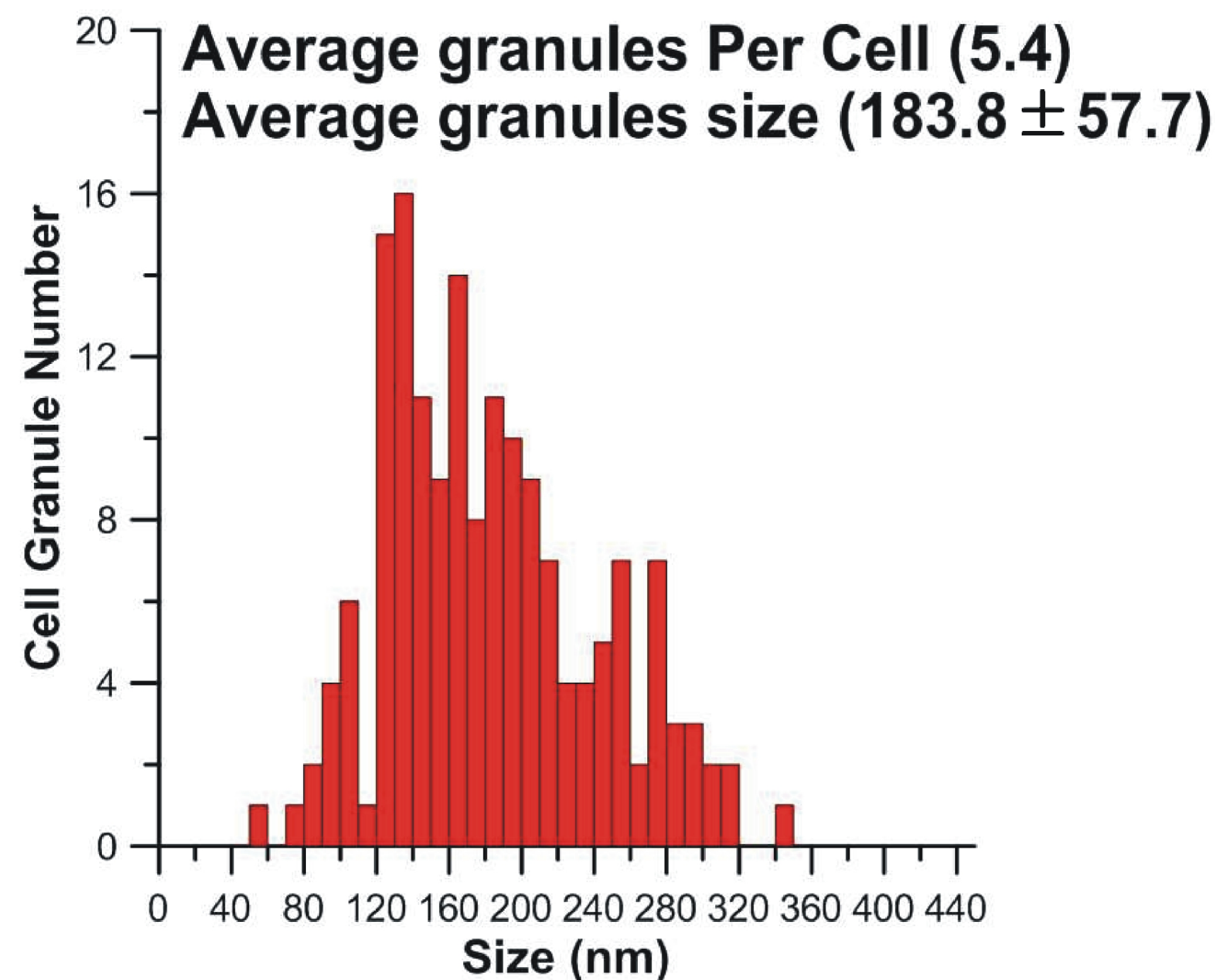

**600 J/m<sup>2</sup>**

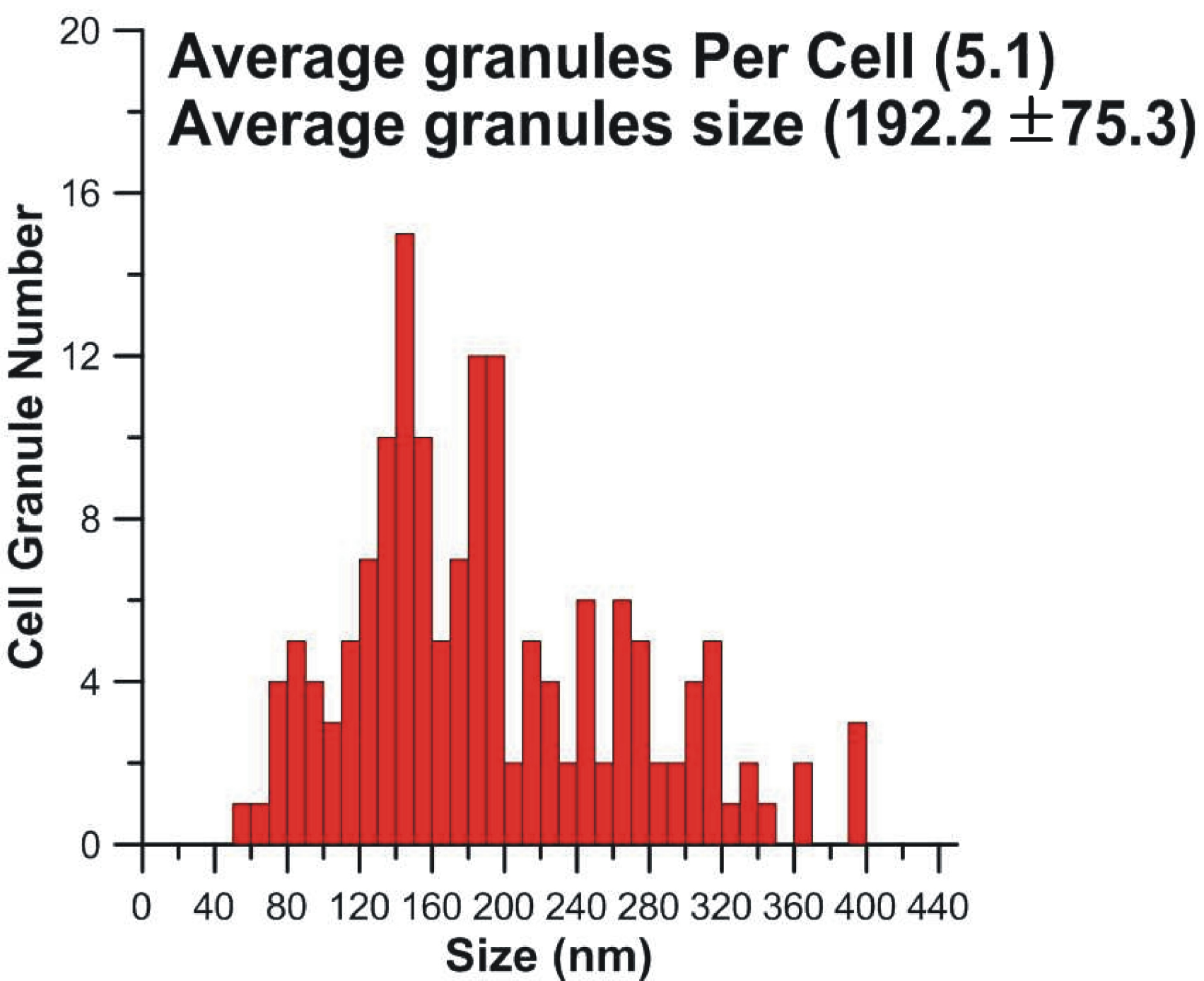

**1800 J/m<sup>2</sup>**

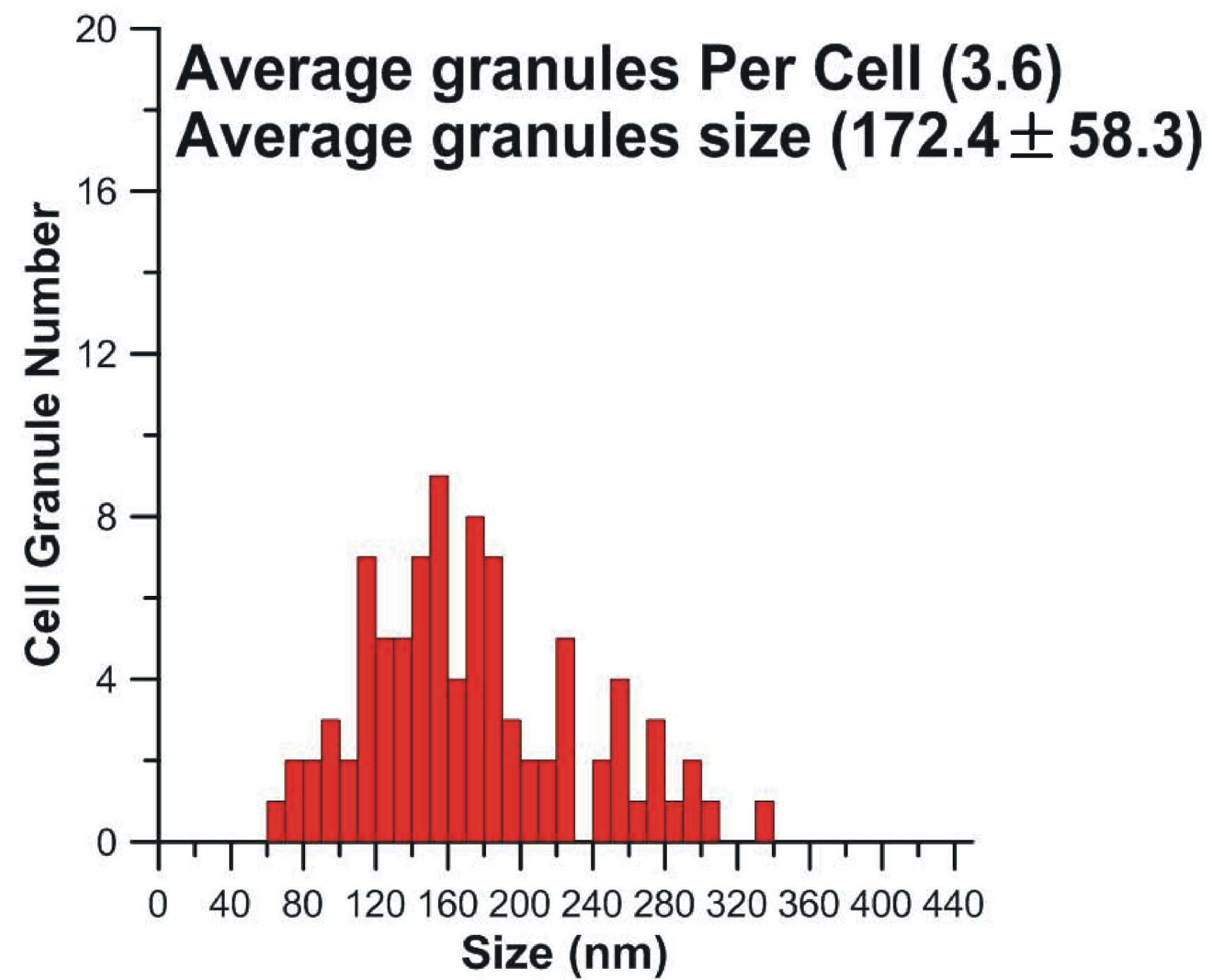

Supplement: Supplementary file 1 [file Presentation1.PDF]
